# An Uncommon Case of Severe Gastric Ulceration Following Radioembolization for Hepatocellular Carcinoma: Clinical Insights and Management Challenges

**DOI:** 10.7759/cureus.94075

**Published:** 2025-10-07

**Authors:** Saad Aldosari, Ahmad A Alsolmi, Abdullah Alsulami, Nawaf Halabi, Fatimah Alturkistani

**Affiliations:** 1 Department of Internal Medicine, Gastroenterology Section, King Abdulaziz Medical City, King Abdullah International Medical Research Center, Jeddah, SAU; 2 Department of Internal Medicine, King Abdulaziz Medical City, King Abdullah International Medical Research Center, Jeddah, SAU; 3 Department of Anatomical Pathology, King Abdulaziz Medical City, King Abdullah International Medical Research Center, Jeddah, SAU

**Keywords:** gastric ulcer, hepatocellular carcinoma, microspheres, radioembolization, saudi arabia, trans-arterial radioembolization, yttrium-90

## Abstract

Yttrium-90 trans-arterial radioembolization (TARE) is a locoregional therapy performed in select patients with hepatocellular carcinoma (HCC). Although generally well tolerated, the procedure can occasionally result in serious complications.

We report the case of a 72-year-old Saudi woman with hypertension, hepatitis C-related cirrhosis, and recurrent HCC who underwent TARE after unsuccessful radiofrequency ablation. Following the procedure, she developed progressive epigastric pain and reduced oral intake. Endoscopy revealed a large gastric ulcer, and histopathology confirmed the presence of yttrium-90 microspheres within the mucosa, consistent with radiation-induced injury. Despite treatment with proton pump inhibitors and antacids, her symptoms persisted and required hospital admission.

Her condition subsequently deteriorated with persistent ulceration and worsening liver function, leading to decompensation, sepsis, and death.

This case illustrates a rare but severe adverse event following TARE and emphasizes the importance of careful preprocedural assessment and close monitoring. Clinicians should maintain vigilance for gastrointestinal complications in patients presenting with abdominal symptoms after TARE to allow timely recognition and management.

## Introduction

Liver malignancy is a major global health concern and its incidence is increasing. Estimates indicate that by 2025, more than one million new cases of liver cancer will be diagnosed annually [1]. Primary liver cancer, mainly hepatocellular carcinoma (HCC), which accounts for 75%-85% of these cases, is the sixth most commonly diagnosed cancer and the third leading cause of cancer-related deaths globally, with approximately 906,000 new cases and 830,000 deaths reported in 2020 [2]. According to the Saudi Health Council, liver cancer is the seventh most prevalent cancer among Saudi men and the tenth most prevalent among Saudi women. In 2020, 450 cases were reported, representing 3.2% of all cancer diagnoses among Saudi nationals, with a median age at diagnosis of 65 years [3].

Intermediate-stage HCC (according to the Barcelona Clinic Liver Cancer Staging Classification) presents with a wide array of tumor sizes, quantities, and varying degrees of liver function, all of which influence patient outcomes after trans-arterial chemoembolization (TACE). A novel approach, trans-arterial radioembolization (TARE), uses the β-ray emitting radioactive isotope yttrium-90 (90Y), known for its short half-life and shallow penetration depth, to provide a powerful radiation-based anti-cancer treatment while causing minimal vessel obstruction [4]. In the treatment of liver tumors, radioembolization (RE) is a widely accepted form of selective internal radiation therapy (SIRT). It delivers a high dose of targeted radiation directly to the blood supply of the tumor via the arterial route, thereby conserving most healthy liver tissue [5]. Even with such precision, complications can emerge if the microspheres inadvertently affect other organs or if radiation-induced damage extends beyond the liver [6]. Side effects can range from general constitutional symptoms, such as fever, to more severe conditions, such as cholecystitis, pancreatitis, radiation pneumonitis, hepatic failure, and severe gastrointestinal ulcers [7].

Gastrointestinal ulceration, in particular, represents a potentially severe complication. While recognized in the literature, its true incidence remains uncertain. It is often underreported and there is limited consensus on optimal management strategies. Herein, we present the case of a 72-year-old Saudi woman who developed severe gastric ulceration after TARE for HCC. To our knowledge, this represents the first case of its kind reported in Saudi Arabia. Beyond its rarity, this report highlights the diagnostic challenges encountered, describes the management undertaken, and reflects on the broader clinical lessons for practice.

## Case presentation

**Figure 1:**
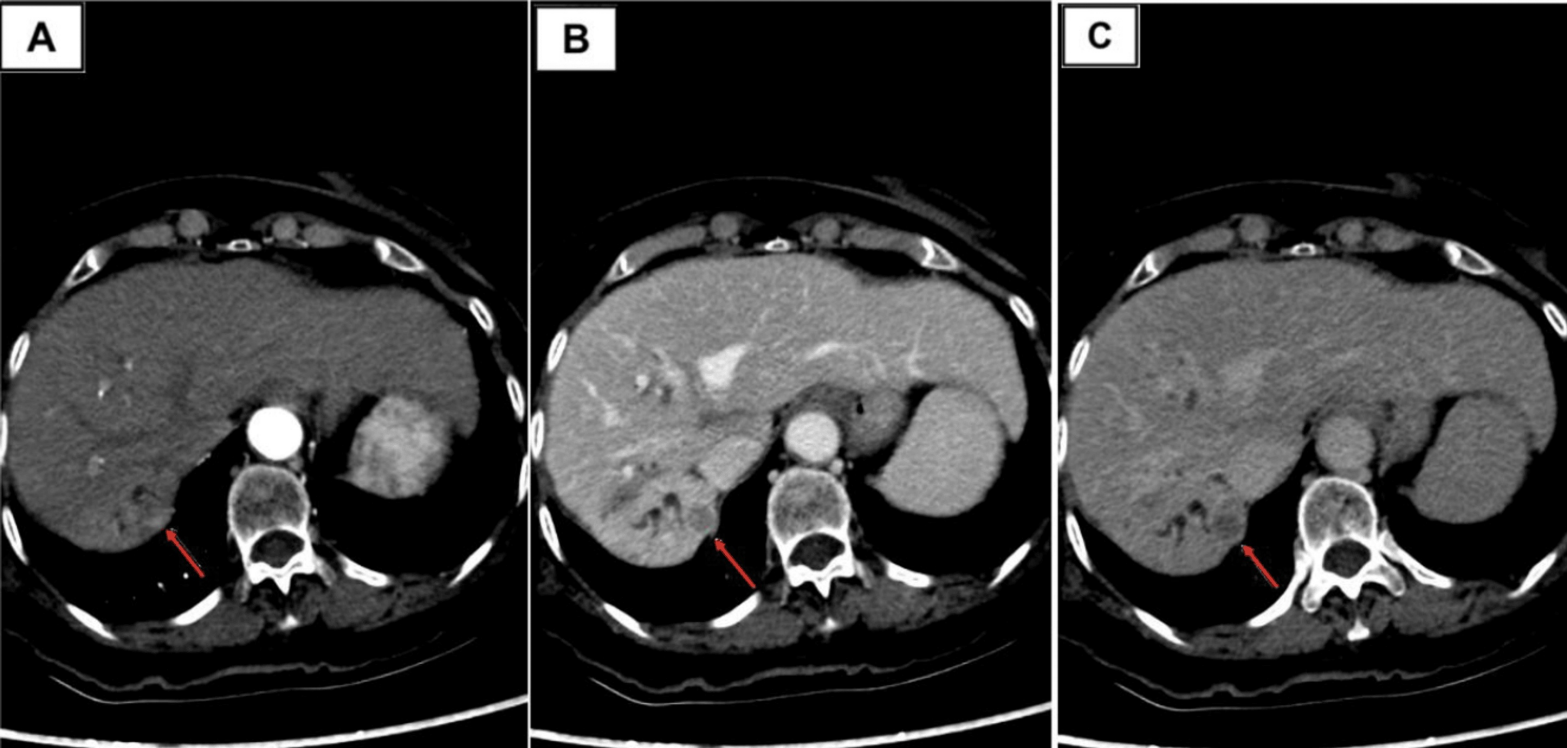
Triphasic liver protocol CT with IV contrast demonstrating an arterially enhancing lesion in hepatic segment VII (red arrows). (A) Arterial phase image shows a subtle hyperenhancing focus measuring 11 mm. (B) In the portal venous phase, the lesion becomes hypoattenuating relative to the background liver, consistent with washout. (C) Delayed phase demonstrates persistent washout with a thin peripheral pseudocapsule. The overall enhancement pattern is diagnostic for hepatocellular carcinoma (LI-RADS 5). LI-RADS: The Liver Imaging Reporting and Data System.

A 74-year-old Saudi woman with a history of hypertension, chronic hepatitis C complicated by cirrhosis (Child-Pugh class B) and HCC underwent radiofrequency ablation in 2015, followed by the recurrence of HCC requiring repeat ablation in 2021. On follow-up, abdominal computed tomography (CT) revealed a new 11 mm lesion in segment VII, categorized as LI-RADS 5 (the Liver Imaging Reporting and Data System 5) (Figure 1). The case was discussed at a multidisciplinary tumor board, and TARE was selected as the next treatment option.

Pre-procedural arterial mapping was performed using Technetium-99m macroaggregated albumin (Tc-99m MAA, 5 mCi) via standard right femoral artery access. Post-mapping single-photon emission computed tomography and computed tomography (SPECT-CT) showed expected tracer distribution with diffuse uptake in the right hepatic lobe, more intense uptake in the known hepatic lesion, no gastrointestinal uptake, and an acceptable lung shunt fraction (5.3%). Two weeks later, the patient underwent an uneventful TARE with Y90 infusion into the right hepatic artery and was discharged without complications.

Twenty days after TARE, the patient presented repeatedly to the emergency department with progressive burning epigastric pain, decreased appetite, and reduced oral intake, managed conservatively with intravenous fluids and analgesia. Symptoms persisted, and she was subsequently evaluated in the gastroenterology clinic. On presentation, vital signs were blood pressure 143/93 mmHg, heart rate 118 beats/min, respiratory rate 19 breaths/min, and oxygen saturation 99%. Laboratory investigations compared with pre-procedure values are summarized in Table 1.

**Table 1:** Laboratory findings upon presentation in comparison to previous values prior the procedure

Related Tests	Current Value	Previous Value	Reference Range
Hemoglobin	11.3 g/dL	11.7 g/dL	11.5-16.5 g/dL
White blood cell count (WBC)	7.5	6.9	4.5-11.0x10^9^/L
Platelets	257	282	150-450x10^9^/L
International normalized ratio (INR)	1.1	1.2	0.8-1.2
Prothrombin time (PT)	12	15	11-14 seconds
Partial thromboplastin time (PTT)	29	28	26-41
Bilirubin Total	15.1	11	2.1-15.5
Gamma-glutamyl transferase (GGT)	27 IU/L	32 IU/L	7-30 IU/L
Alkaline phosphatase (ALP)	126 U/L	73 U/L	39-114 U/L
Aspartate transaminase (AST)	25 U/L	20 U/L	5-34 U/L
Alanine transaminase (ALT)	20 U/L	7 U/L	6-28 U/L

**Figure 2:**
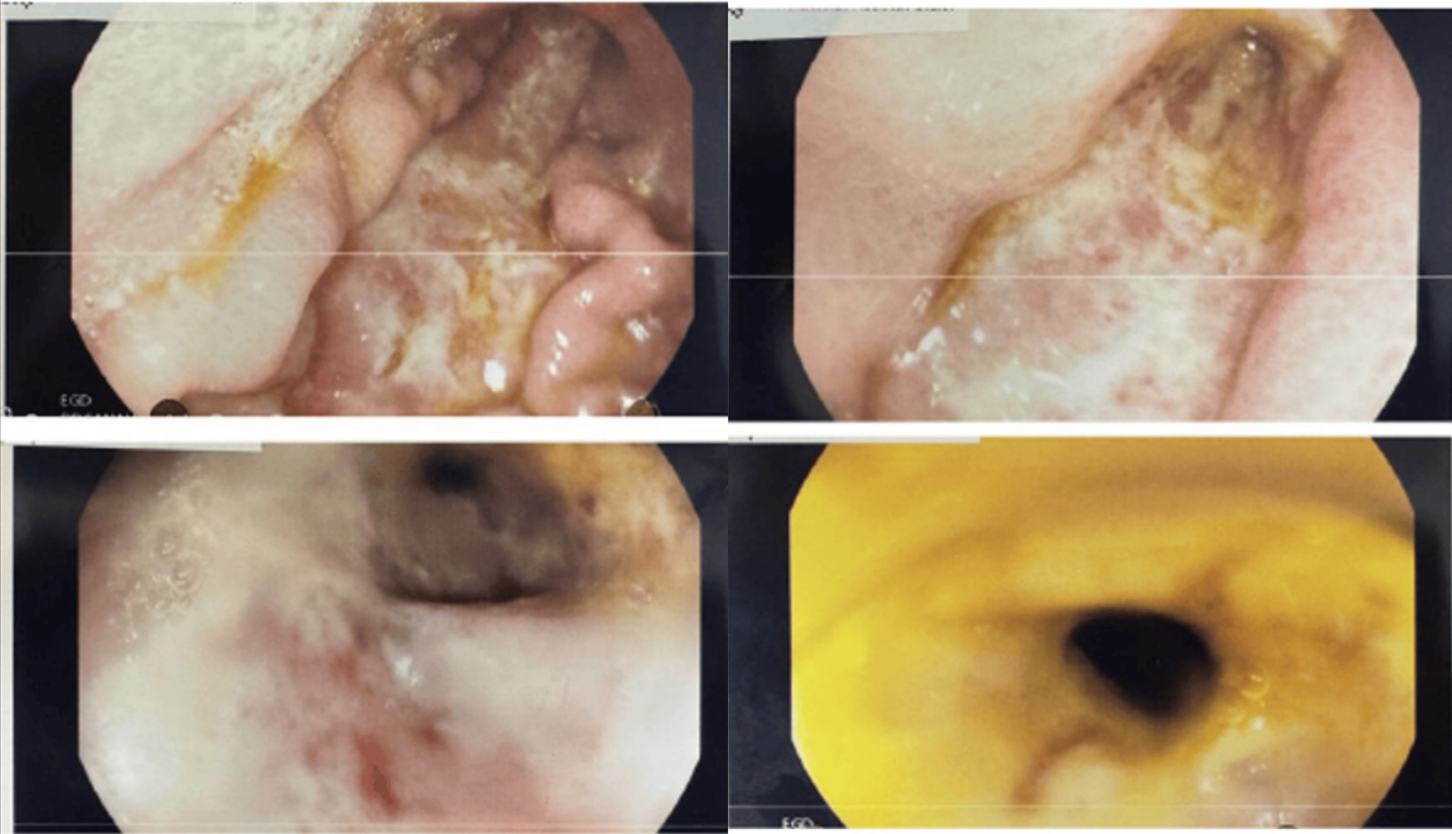
Esophagogastroduodenoscopy (EGD) showing severe long gastric ulcer with circumferential narrowing.

**Figure 3:**
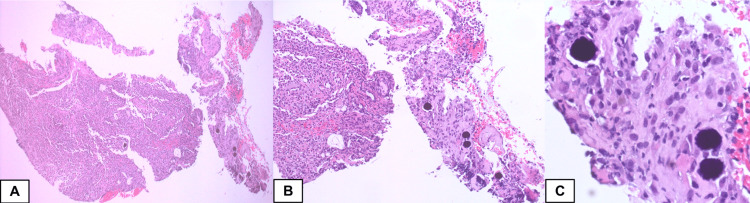
Endoscopic biopsy obtained from the stomach. Hematoxylin and eosin stain Endoscopic biopsy obtained from the stomach showed two fragments of gastric mucosa with ulceration, acute inflammation and granulation tissue formation (A, low magnification view ×40). Purplish microspheres are seen within the stroma, those are rounded particles with uniform size and shape (B and C, Intermediate and high magnification view, ×200 and ×400 respectively).

**Figure 4:**
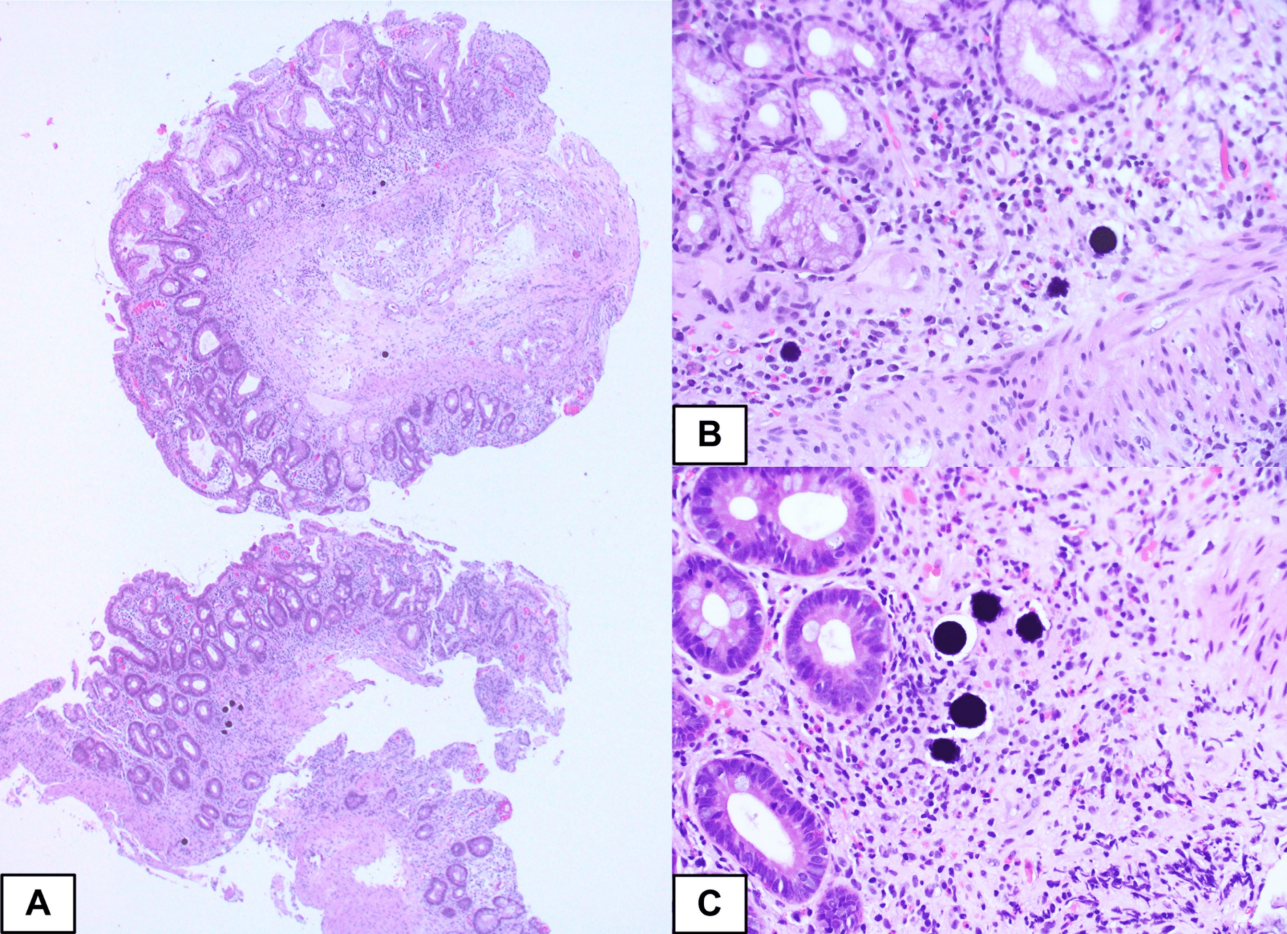
Endoscopic biopsy obtained from the duodenum The figure show an endoscopic biopsy obtained from the duodenum showing focal villous blunting (A, low power magnification, ×40). The lamina propria show small mucosal vessels that are lodged by purplish microspheres that have the same size and shape (B and C, intermediate and high power magnification, ×200 and ×400)

An urgent esophagogastroduodenoscopy (EGD) performed the same week demonstrated a severe long gastric ulcer extending from the proximal posterior wall of the greater curvature, becoming circumferential at the antrum with significant luminal narrowing (Figure 2). Passage was not possible with a standard scope and required a pediatric neonatal scope to reach the duodenum. The mucosal transition between stomach and duodenum was indistinct. Biopsies from the stomach and duodenum revealed Y90 microspheres with duodenal villous blunting and no evidence of malignancy (Figures 3,4). A diagnosis of radiation-induced severe gastric ulceration was established, and the patient was admitted for further management.

Initial management included proton pump inhibitors, sucralfate, antacids, intravenous fluids, and antiemetics. Despite this regimen, symptoms persisted and oral intake remained poor, necessitating hospital readmission. Nasojejunal feeding was proposed, but the patient declined, and total parenteral nutrition (TPN) was initiated instead. Despite these measures, symptoms continued to progress, and repeat endoscopy showed persistent, severe ulceration with pyloric narrowing.

During the same admission, the patient’s cirrhosis decompensated, with worsening ascites, encephalopathy, and a decline from Child-Pugh class B to class C. Given her age and high operative risk, surgical intervention was deemed unsuitable. She subsequently developed hypotension and sepsis with disseminated intravascular coagulation, requiring intensive care unit admission, where she later passed away.

In summary, this patient with cirrhosis and HCC developed unusually early and severe radiation-induced gastric ulceration after TARE, refractory to conservative management. The course was further complicated by cirrhosis decompensation, ultimately leading to death. This case highlights the challenges of managing post-TARE gastrointestinal complications in high-risk patients.

## Discussion

HCC is the most common type of liver cancer, originating from hepatocytes, and is often associated with chronic liver diseases such as cirrhosis and hepatitis B or C. TACE and TARE are treatment options for managing HCC. TACE involves the direct delivery of chemotherapy to the liver tumor, followed by the administration of embolic agents to block blood flow, thereby enhancing the effectiveness of chemotherapy. In contrast, TARE, also known as SIRT, uses microspheres loaded with 90Y to target tumors with focused radiation therapy [8]. The cytotoxic effect of radioembolization arises primarily from its radioactive properties rather than ischemia. Another advantage of TARE is its reduced embolic effect compared with TACE, which makes it suitable for patients with main portal vein thrombosis.

Additionally, TARE has been associated with a longer time to progression and lower toxicity than TACE [9-11]. Comparable to other radiological interventions, trans-arterial treatments are typically safe but can lead to adverse events. Although most of these complications are minor, some can result in substantial morbidity and mortality, including gastrointestinal ulcerations. The reported incidence of this condition ranges from 2.9% to 4.8% [12].

Gastrointestinal ulceration from TARE arises from the inadvertent delivery of microspheres to the stomach and duodenum via the anastomotic blood supply to the GI tract. Various causes have been identified, including dynamic changes in Y90 microspheres, undetected collateral blood supply or formation of new collateral arteries, and vessel spasms induced by catheter stimulation during the procedure [13]. Two principal mechanisms have been proposed to explain the resulting tissue injuries: direct cytotoxic effects of radiation on gastric and intestinal tissues, and tissue ischemia induced by occlusion of the arterial supply to these organs. Several studies suggest that direct radiotoxicity is the predominant cause [14,15].

To minimize such complications, a preassessment protocol is recommended before TARE. The institutional SIRT protocol comprises several critical procedures to ensure precise and safe therapeutic delivery. Initially, a dual-phase CT scan is performed to delineate the tumor anatomy. This is followed by hepatic angiography to identify hepatic artery branches and collateral vessels supplying the GI tract and the tumor. To mitigate radiation-induced GI injury, coil embolization is performed on the gastroduodenal artery and other GI collateral branches of the hepatic artery. Subsequently, technetium-99 macroaggregated albumin is administered into the hepatic artery branch perfusing the tumor. Planar scintigraphy and SPECT-CT are used to detect extrahepatic shunting in the GI tract or pulmonary system [16]. In our case, a similar protocol was followed.

Despite adherence to protocol and an acceptable lung shunt fraction, our patient developed unusually severe and refractory gastroduodenal ulceration. Possible contributors include cirrhosis-associated vascular alterations that may have facilitated unintended microsphere deposition, as well as advanced age and comorbidities that increased tissue vulnerability. These factors may help explain why the complication occurred in such a profound form despite adherence to standard protocols.

Clinical manifestations of TARE-induced gastrointestinal ulceration include abdominal pain, nausea, and vomiting. In the present case, the patient presented with these symptoms, which progressed to anorexia and reduced oral intake, resulting in unintentional weight loss. Such manifestations are nonspecific and resemble many other gastrointestinal diseases, often leading to diagnostic delay. Furthermore, the average time to symptom onset described in the literature is four months [12]. In our patient, however, symptoms began only 20 days after the procedure, representing an unusually early onset. Endoscopy remains the cornerstone of diagnosis, and biopsy confirmation of Y90 microspheres is pathognomonic. In this patient, endoscopic and histopathological findings were consistent with the diagnostic features described in the literature [15].

Management of radiation-induced gastric ulcers is typically conservative, involving proton pump inhibitors, antacids, and antiemetics [5,6,14]. Our patient received such treatment, but symptoms persisted. Nasojejunal feeding has been described as beneficial in similar cases [5], but in this instance, the patient declined, necessitating the initiation of total parenteral nutrition. Despite these measures, symptoms progressed, and repeat endoscopy revealed severe refractory ulceration with pyloric narrowing. Surgical intervention has been reported as a definitive therapy in refractory cases, including subtotal gastrectomy [6]. However, in our patient, advanced cirrhosis and age rendered surgery high risk, leaving conservative management as the only feasible option.

The patient’s advanced cirrhosis further limited management options and likely contributed to her deterioration. In the reviewed literature, Brock et al. reported the onset of new cirrhosis shortly after SIRT for hepatocellular carcinoma, concluding that cirrhosis and liver failure may be potential complications requiring close monitoring [17]. Our case adds to this concern by demonstrating an unusually early onset of ulceration, a refractory course despite correct protocol adherence, and a fatal outcome. These findings underscore the importance of closer post-TARE surveillance in high-risk patients, early recognition of nutritional decline, and timely multidisciplinary involvement. Beyond its rarity as the first case reported in Saudi Arabia, this case highlights the need for improved risk stratification, standardized surveillance, and systematic reporting of such complications to inform best practices and optimize patient outcomes.

## Conclusions

In conclusion, this case illustrates that severe gastroduodenal ulceration can occur after hepatic radioembolization despite correct mapping and strict adherence to established protocols. To our knowledge, it also represents the first such case reported in Saudi Arabia. Its clinical course was particularly notable for the unusually early onset of symptoms (20 days compared with an average of four months), the refractory progression despite guideline-based management, and the ultimately fatal outcome. These features underscore the importance of closer post-TARE surveillance in elderly and cirrhotic patients, timely recognition of nutritional decline, and early multidisciplinary intervention, including consideration of alternative strategies when conservative measures fail. Beyond its regional novelty, this case highlights the need for improved risk stratification, standardized surveillance protocols, and systematic reporting of such complications to inform best practices and optimize outcomes.
